# Empowering artificial intelligence in characterizing the human primary pacemaker of the heart at single cell resolution

**DOI:** 10.1038/s41598-024-63542-6

**Published:** 2024-06-18

**Authors:** Alexandru Chelu, Elizabeth J. Cartwright, Halina Dobrzynski

**Affiliations:** 1 Division of Cardiovascular Sciences, Faculty of Biology, Medicine and Health, The University of Manchester, Manchester, M13 9PL UK; 2 Department of Anatomy, Jagiellonian University Medical College, 31-008 Kraków, Poland

**Keywords:** Bioinformatics, RNA sequencing, Machine learning, Cardiovascular biology

## Abstract

The sinus node (SN) serves as the primary pacemaker of the heart and is the first component of the cardiac conduction system. Due to its anatomical properties and sample scarcity, the cellular composition of the human SN has been historically challenging to study. Here, we employed a novel deep learning deconvolution method, namely Bulk2space, to characterise the cellular heterogeneity of the human SN using existing single-cell datasets of non-human species. As a proof of principle, we used Bulk2Space to profile the cells of the bulk human right atrium using publicly available mouse scRNA-Seq data as a reference. 18 human cell populations were identified, with cardiac myocytes being the most abundant. Each identified cell population correlated to its published experimental counterpart. Subsequently, we applied the deconvolution to the bulk transcriptome of the human SN and identified 11 cell populations, including a population of pacemaker cardiomyocytes expressing pacemaking ion channels (*HCN1, HCN4, CACNA1D*) and transcription factors (*SHOX2* and *TBX3*). The connective tissue of the SN was characterised by adipocyte and fibroblast populations, as well as key immune cells. Our work unravelled the unique single cell composition of the human SN by leveraging the power of a novel machine learning method.

## Introduction

The cardiac conduction system orchestrates the electrical activity of the heart, with the sinus node (SN) located in the right atrium serving as the primary pacemaker^1^. The SN is a small, heterogenous and compartmentalized structure of the myocardium that initiates the action potential and controls the heart rate^1–3^. The joint effect of Ca^2+^ regulators and ion channels contribute to the pacemaking activity within the SN^2,4^.

Single cell RNA sequencing (scRNA-Seq) has paved the way to unravel the cellular heterogenicity within the cardiac tissue^5,6^. The cellular landscape of the SN has been characterised across several mammalian species^7^, with mice being the most used animal model^7,8^. It is now evident that the initiation and propagation of the electrical activity within the SN originates from a subpopulation of cardiomyocytes, which express key conduction genes and transcription factors^7–9^. Moreover, the abundant extracellular matrix found in the SN^1^ is thought to isolate the electrical activity of the pacemaking cardiomyocytes from their surrounding atrial cells^10–12^. The cellular heterogeneity of the SN includes fibroblasts, adipocytes and macrophages, residing in compartmentalised areas^8,9^. However, our knowledge of the cellular composition of the SN at single cell resolution is currently limited to animal models^5,7,8,13^, with only one study performed in humans^9^.

Ongoing adoption of scRNA-Seq and other ‘omics methods is exponentially increasing the wealth of publicly available biological data. Concurrently, new computational tools are emerging as novel solutions to interrogate new and existing data using machine learning techniques^14,15^. Recently, a deep learning deconvolution algorithm, namely Bulk2Space^16^, was introduced as a powerful tool to resolve the transcriptional heterogeneity of bulk RNA-Seq data into scRNA-Seq. Thus, existing bulk transcriptomes can be further analysed to reveal unprecedented biological insights. Importantly, this tool allows to study the single cell composition of any tissue of interest virtually for free, as the experimental work and costs associated are lifted. Here, we employed Bulk2Space, originally developed by Liao et al.^16^, to characterise the single cell landscape of the human SN using a mammalian non-human reference dataset. As a proof of concept we evaluated the deconvolution performance on a well-defined cardiac tissue, the right atrium. We deconvoluted human bulk RNA-Seq of the right atrium using publicly available mouse scRNA-Seq data^17^ as reference and successfully recapitulated the key cardiac cells of the human atrium. We then applied this method to deconvolute human bulk RNA-Seq data of the SN, unravelling populations of pacemaking and non-pacemaking cardiomyocytes. We have also identified fibroblasts, adipocytes, macrophages and lymphoid cells as the main non-myocyte populations occupying the surrounding nodal area rich in connective tissue. Our findings confirmed that the human SN is a highly compartmentalised tissue, with the pacemaking cardiomyocytes being the focal centre of electrical activation and potentially being supported by a plethora of non-myocyte cells. Our study has demonstrated how artificial intelligence (A.I.) can be employed to unravel new insights in human cardiac biology using non-human models. Furthermore, our biologically accurate cellular characterization of the SN demonstrated how A.I. has the potential to shed light into previously challenging tissues and could be extended to characterise other heart regions such as the atrioventricular node, as well as other tissues of the human body.

## Results

### The single cell composition of the human right atrium

**Figure 1:**
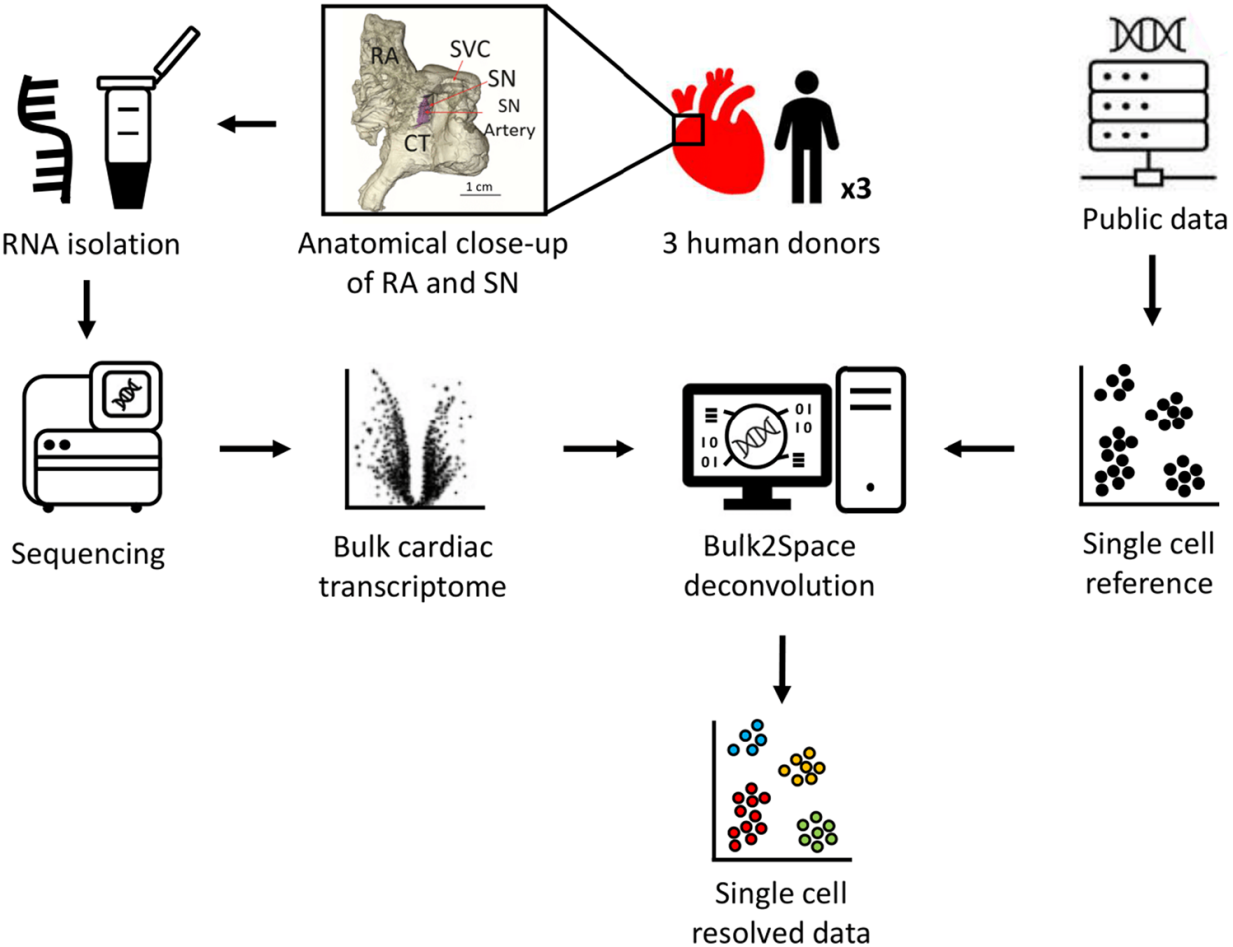
Deconvolution of bulk RNA-Seq. Atrial and SN tissues were obtained and dissected from the same hearts of 3 human donors. Anatomical structure of RA and SN were adapted from Petkova et al.^23^. RNA isolation and sequencing was performed as described by our group^20,23^. Bioinformatics data processing generated the bulk cardiac transcriptome of the human right atrium. Through publicly available data, a murine right atrium scRNA-Seq dataset was retrieved and used as training reference for Bulk2Space. Finally, the bulk cardiac transcriptome of the human atrium was resolved into biologically feasible single cell data. SVC, superior vena cava; CT, crista terminalis.

Bulk2Space was designed as a deconvolution tool to generate single cell transcriptomes from bulk RNA-Seq data using publicly available scRNA-Seq datasets as a training reference^16^. To do so, the reference must be biologically similar to the bulk RNA-Seq data to be deconvoluted. The authors demonstrated robust results in deconvoluting bulk RNA-Seq data using scRNA-Seq references from the same species, however the performance is unknown when using a different yet related mammalian species. Given that cell identity can be defined by the clustering patterns observed in single-cell gene expression profiles^18^, if the expression features demonstrate remarkable stability across various conditions, technologies, and species^19^, then one may use a related mammalian species scRNA-Seq data to deconvolute human bulk RNA-Seq data. Based on this assumption, we assessed whether Bulk2Space can deconvolute our in-house RNA-Seq data of the human right atrium^20^ using right atrium scRNA-Seq data from mice^17^ as training reference (Fig. 1). We chose Tabula Muris as the reference due to its comprehensive and accurate representation of the murine cell biology across tissues^17^. From the cardiac dataset, we filtered 2899 atrial cells.

**Figure 2:**
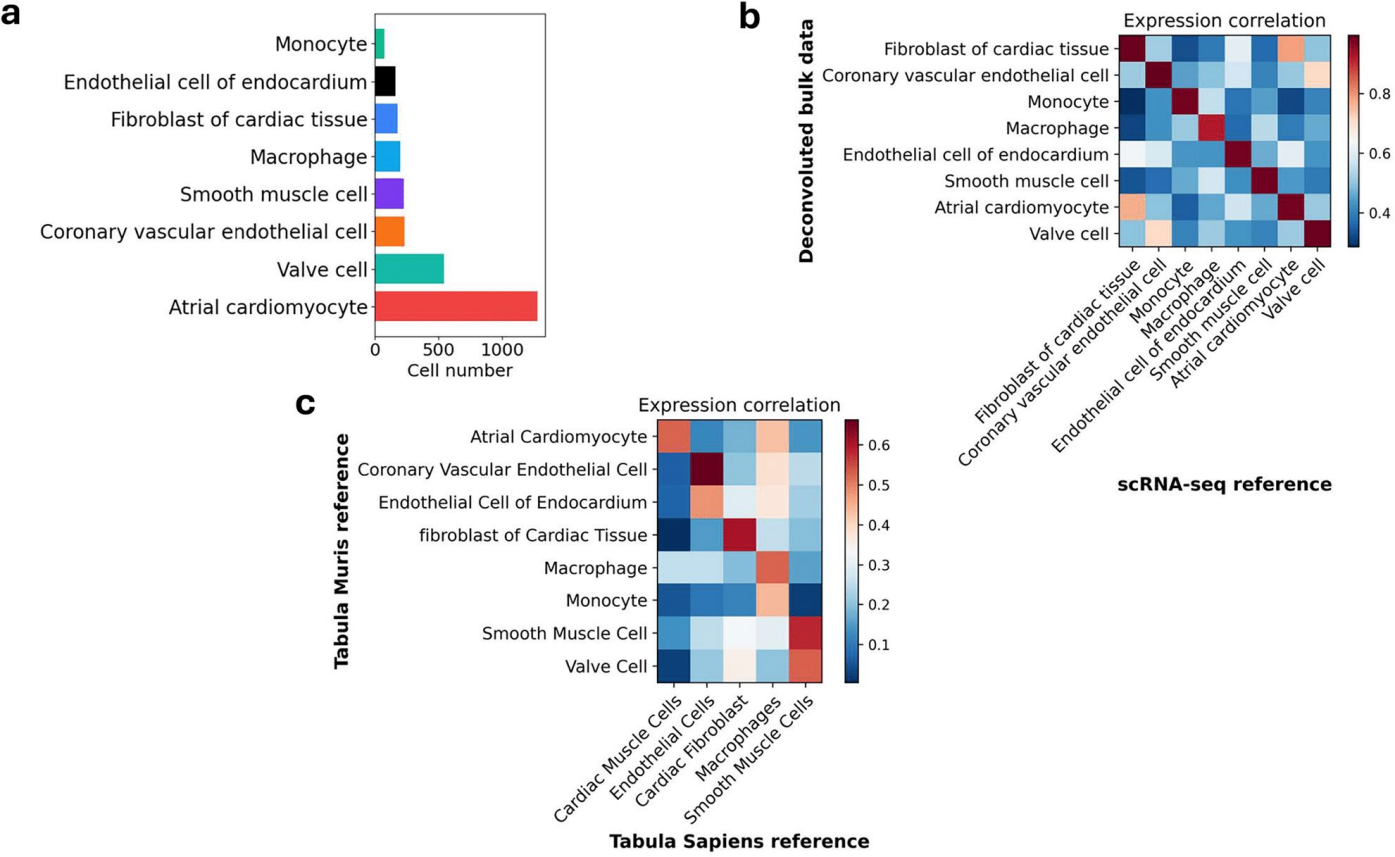
Deconvoluted single cells of the human right atrium. (**a**) Cell type proportions from the human right atrium data generated by Bulk2Space. Eight broad cell types were identified by the algorithm. (**b**) Pairwise expression correlation of cell type specific marker genes between single cells generated by Bulk2Space and the scRNA-Seq reference for mouse right atrium. (**c**) Pairwise expression correlation of cell type specific marker genes between single cells generated using a mouse and human scRNA-Seq references (Tabula Muris and Tabula Sapiens). Marker genes were calculated by the ‘rank_genes_groups’ function in Scanpy. P value was calculated with the Wilcoxon rank-sum test.

We converted mouse genes into their human counterpart using orthologs, with a 66% conversion rate. A matrix of 15,248 genes and 2899 cells was used to train the model and subsequently, deconvolute the bulk human right atrium transcriptome. Bulk2Space successfully resolved the bulk RNA-Seq into single cell profiles and identified eight cell types, with cardiac myocytes being the most abundant cell type in the human right atrium (Fig. 2a). We correlated the expression of marker genes specific to each cell type to their experimental counterpart and found a strong correlation (Fig. 2b). Additionally, we validated the accuracy of results generated using a mouse reference by deconvoluting the bulk data with a human reference, namely Tabula Sapiens^21^. Cell types generated using different training datasets showed a strong correlation (Fig. 2c). These results demonstrated that generated cells from the human atrium are biologically feasible and comparable to those of mice.

**Figure 3:**
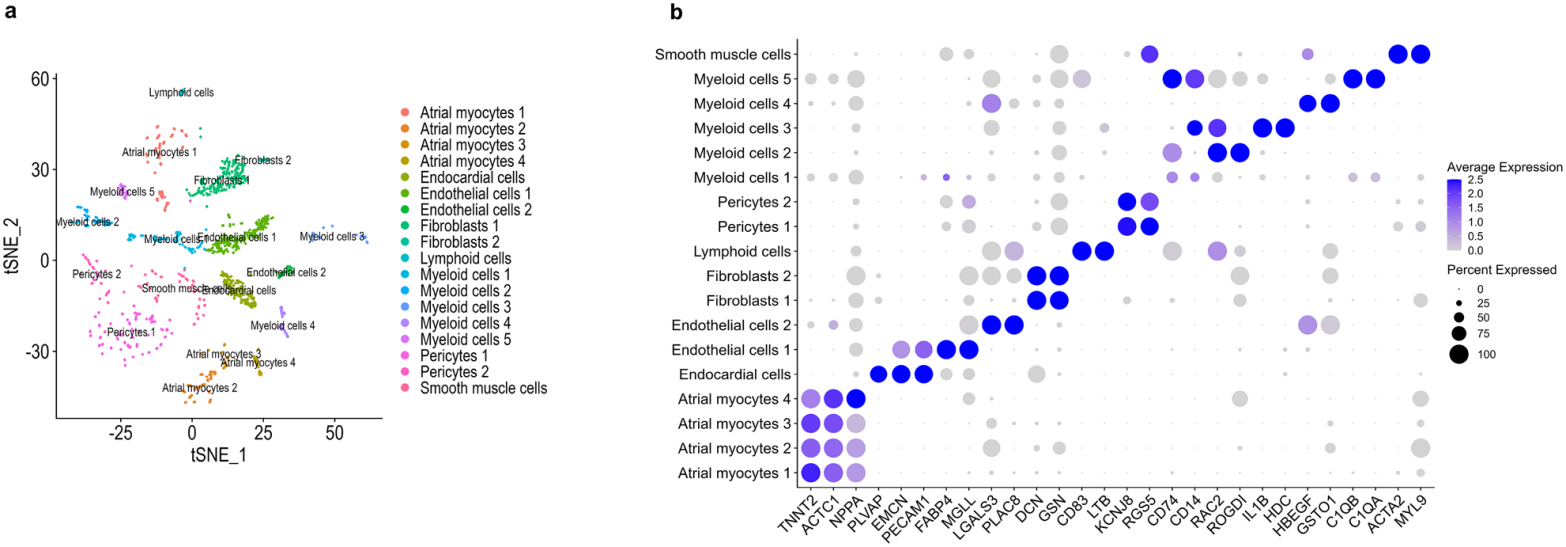
Characterising of the human right atrium at single cell resolution. (**a**) Two-dimensional t-distributed stochastic neighbour embedding (tSNE) plot showing all cell subpopulation of the human right atrium. Each dot is an individual cell and is coloured according to the cluster it belongs to. (**b**) Dotplot showing the expression (log_2_FC) of selected marker genes in atrial myocytes, endocardial cells, fibroblasts, pericytes, leukocytes and smooth muscle cells.

To further validate the cellular composition of the human right atrium, we analysed the data using a standard scRNA-Seq pipeline. Projecting the cells to a two-dimensional t-distributed stochastic neighbour embedding (tSNE) plot allowed us to identify 18 clusters, which corresponded to atrial myocyte, endothelial, fibroblast, pericyte and leukocyte cell subpopulations (Fig. 3a). Each cell subpopulation was manually annotated based on its highly expressed marker genes (Fig. 3b) and cross validated against publicly available studies of the human right atrium^6,22^. In conclusion, we showed that machine learning based deconvolution can be used to generate the human single-cell profiles using non-human references. As a proof of principle, we successfully recapitulated the single cell composition of the human right atrium.

### The characterization of the human sinus node at single cell resolution

As evidenced from our single cell characterization of the human right atrium, Bulk2Space proved to be a robust tool for characterizing the human tissues using mammalian non-human references. Thus, we extended this method to the less well characterised human SN. Specifically, our previously described bulk RNA-Seq data of the human SN^20^ was used for deconvolution. The availability of SN single cell data is currently very limited, with only two studies providing accessible data from mice^7,8^. Liang et al.^7^ performed scRNA-Seq on mouse SN but pooled myocytes from both atrial and ventricular origin, and thus the SN data could not be distinguished from atrial and ventricular data. Linscheid et al.^8^ performed single nucleus RNA-Seq (snRNA-Seq) on mouse SN and isolated the tissue using the same protocol described in the collecting tissue for our bulk RNA-Seq data. Furthermore, their results suggest that their data is more biologically comparable to that from our bulk RNA-Seq. Thus, we used the data from Linscheid et al.^8^ as reference.

**Figure 4:**
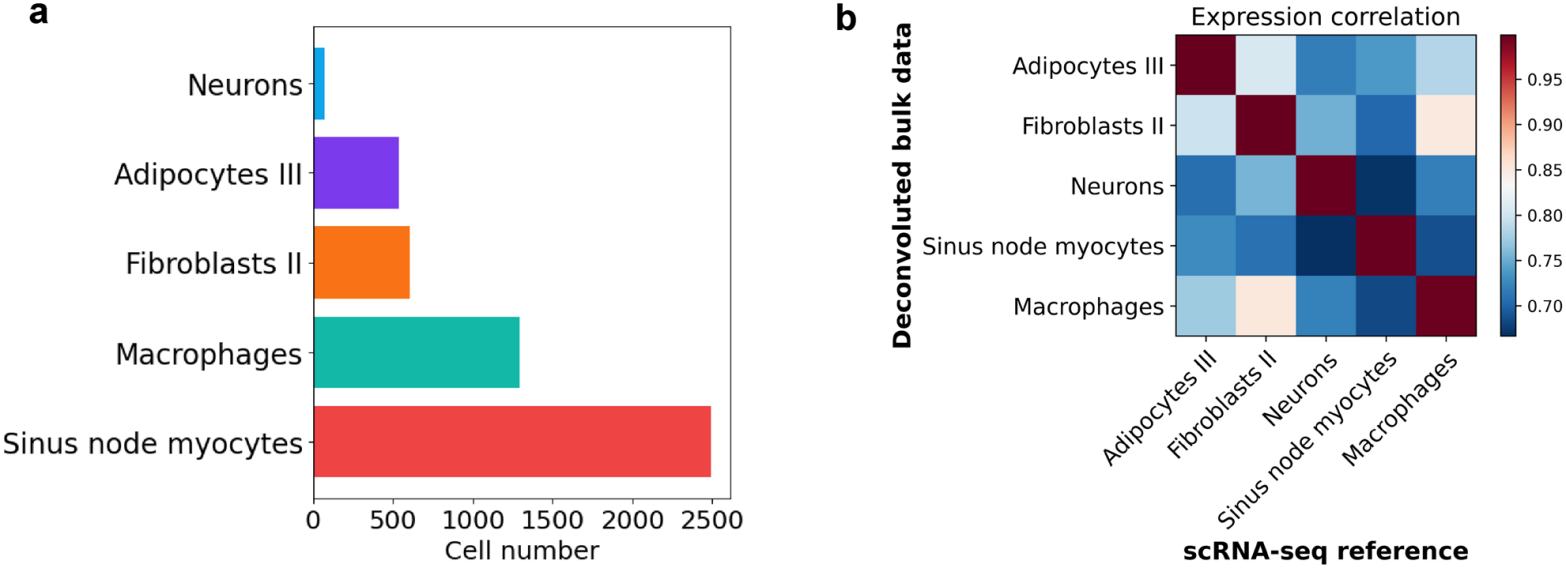
Deconvoluted single cells of the human sinus node. (**a**) Cell type proportions from the human SN data generated by Bulk2Space. Five broad cell types were identified by the algorithm. (**b**) Pairwise expression correlation of cell type specific marker genes between single cells generated by Bulk2Space and the scRNA-Seq reference for mouse SN. Marker genes were calculated by the ‘rank_genes_groups’ function in Scanpy. *P* value was calculated with the Wilcoxon rank-sum test.

**Figure 5:**
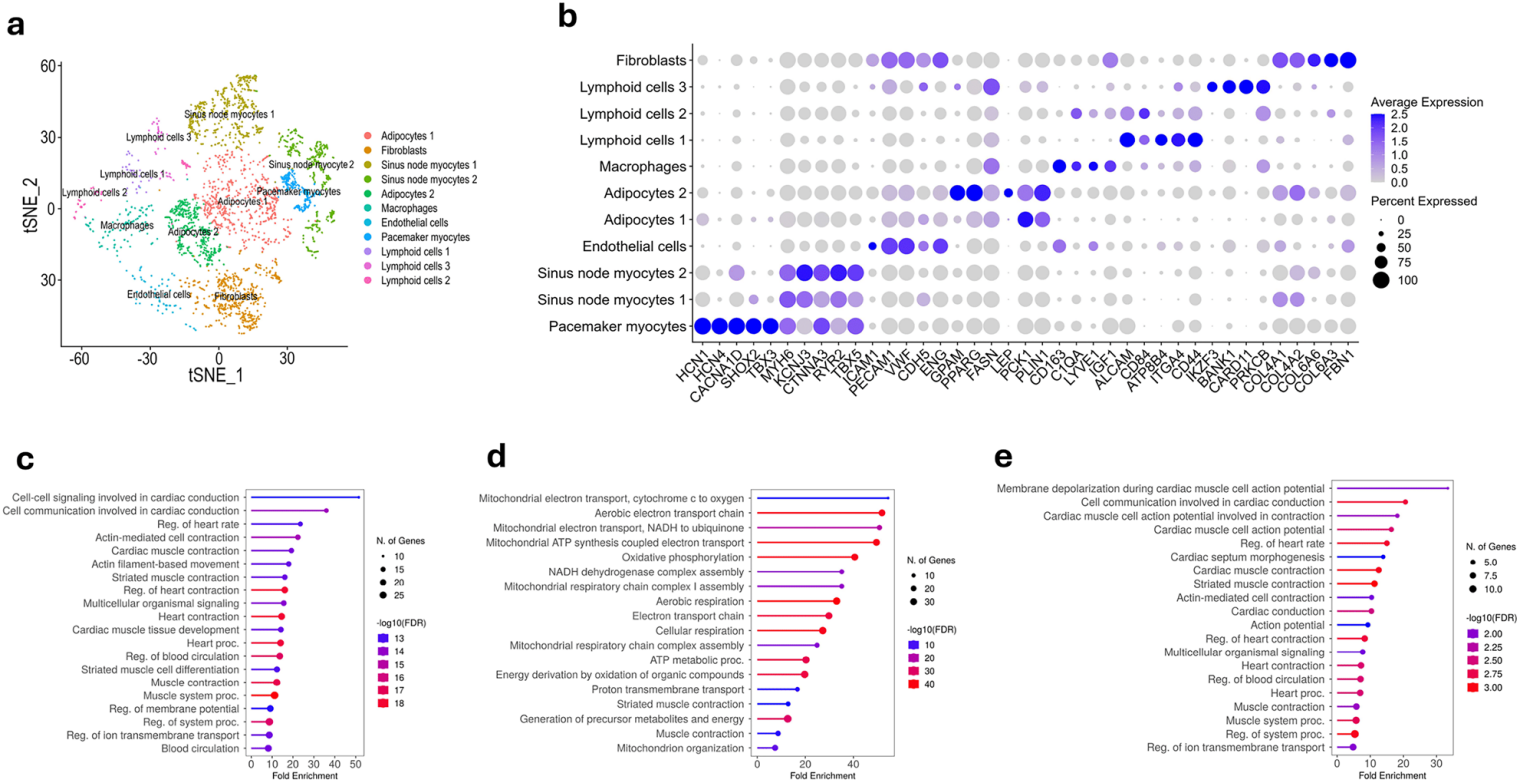
Characterising the human sinus node at single cell resolution. (**a**) Two-dimensional t-distributed stochastic neighbour embedding (tSNE) plot showing all cell subpopulation of the human SN. Each dot is an individual cell and is coloured according to the cluster it belongs to. (**b**) Dotplot showing the expression (log_2_FC) of selected marker genes in SN myocytes, adipocytes, fibroblasts, macrophages, endothelial cells, and lymphoid cells. (**c**) Lollipop plots showing the significantly enriched biological processes in pacemaking myocytes, (**d**) SN myocytes 1, and (**e**) SN myocytes 2 according to Gene Ontology.

We converted mouse genes into their human counterpart using orthologs, with a 58% conversion rate. A matrix of 15,579 genes and 5004 cells was used to train the model and subsequently, deconvolute the bulk human SN transcriptome. SN myocytes were the most abundant cell populations, followed by macrophages, fibroblasts, adipocytes, and a small number of neuronal cells (Fig. 4a). We found that cellular composition of the human SN is biologically similar to that of the mouse SN, as cell specific marker genes highly correlated between the two datasets (Fig. 4b). Further analysis of the human SN single cell data revealed eleven clusters, eight of which corresponded to subpopulations of SN myocytes, adipocytes, and lymphoid cells (Fig. 5a). Beyond expressing well known cardiac myocyte markers such as *MYH6, CTNNA3, RYR2* and *TBX5*^5,8,9,24^, a subpopulation of SN myocytes also expressed key pacemaking ion channel genes such as *HCN1, HCN4, CACNA1D*^9,23^ (Fig. 5b). High expression of well-known conduction transcription factors such as *SHOX2* and *TBX3*^4,20,23^ was also specific to this subpopulation. Thus, we concluded that this subpopulation of myocytes is the key pacemaking unit within the SN, responsible for generating the electrical impulse. Other myocyte subpopulations were found in the SN, expressing contractile genes higher than conduction genes. Furthermore, Gene Ontology enrichment analysis revealed that heart rate and cardiac conduction cell-signalling processes were strongly enriched in pacemaking myocytes (Fig. 5c). We identified distinct biological processes within non-pacemaking myocytes specializing in energetic and conduction propagation roles (Fig. 5d,e). This suggests a novel finding of the functional compartmentalisation of the SN, with pacemaking and non-pacemaking myocytes. Additionally, we identified key biological processes enriched in non-myocytes cells (Supplementary Data 1).

Fibroblasts and adipocytes were the second most abundant non-myocyte cell populations of the human SN. Fibroblasts expressed high levels of *COL4A1, COL4A2, COL6A3* and *COL6A6* which is consistent with previous studies^6,9^. Adipocytes were characterised by expression of *GPAM, PPARG, FASN, PCK1, PLIN1* and low levels of *LEP*^6,8^. The SN has an important immune cell population which is thought to play an important role in the electrophysiology of the tissue^20,24^. Consistent with this, we detected the presence of tissue-resident macrophages expressing *CD163, C1QA, LYVE1* and *IGF1*. Moreover, additional immune cells that we classified as lymphoid cells were found in the human SN.

**Figure 6:**
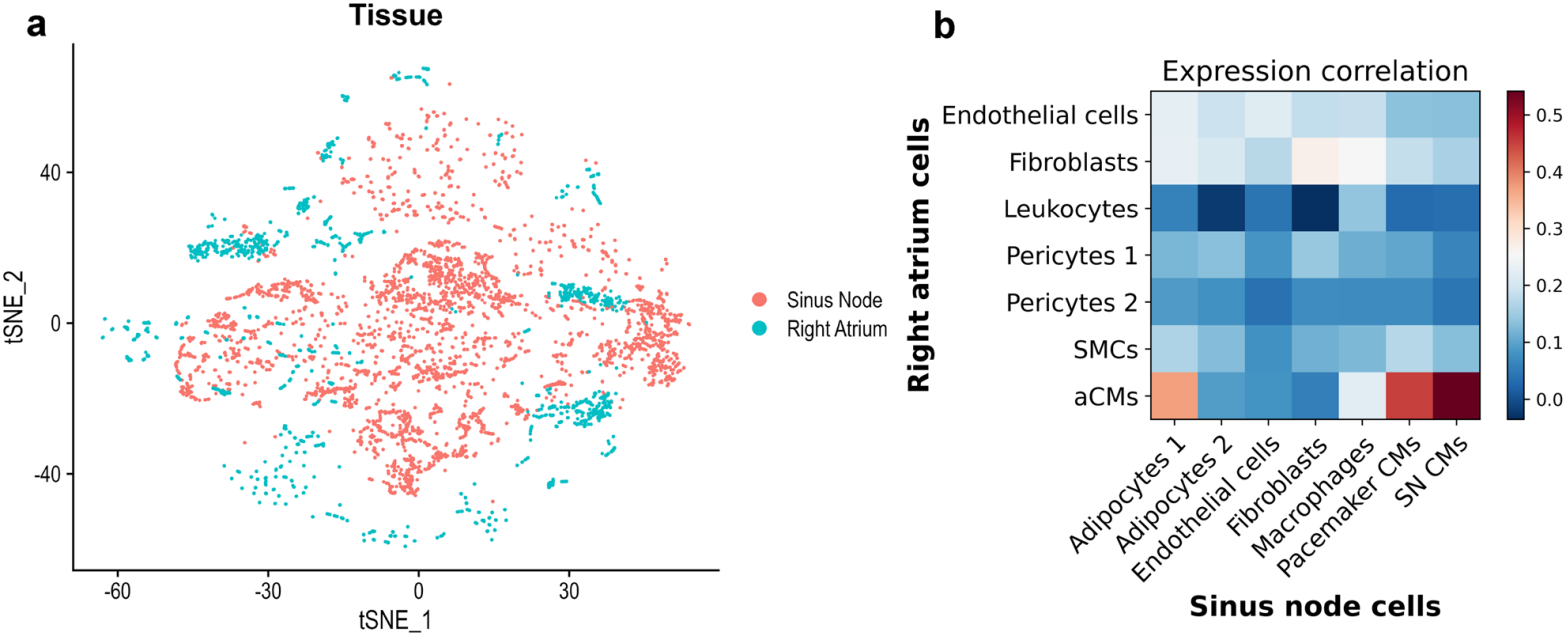
The unique cellular composition of the sinus node. (**a**) Two-dimensional t-distributed stochastic neighbour embedding (tSNE) plot showing the integrated scRNA-Seq datasets of the right atrium and SN, coloured based on tissue type. (**b**) Pairwise expression correlation of cell type specific marker genes between cells of the right atrium and SN. Marker genes were calculated by the ‘rank_genes_groups’ function in Scanpy. *P* value was calculated with the Wilcoxon rank-sum test.

The SN can be viewed as a small yet specialised niche within the right atrium orchestrating the initiation and propagation of the action potentials. Our data showed mutual cell types within the human right atrium and the SN; however, it is unclear whether similar cell types are also biologically similar. To address this, we explored the cellular diversity of the human SN. We integrated the SN scRNA-Seq data with that of the right atrium using canonical correlation analysis^25^. Upon integration, we found that the cells of the SN minimally overlap with those of the right atrium (Fig. 6a). Despite the presence of endothelial cells, fibroblasts and immune cells in both tissues, we found that these cells poorly correlate with each other across tissues (Fig. 6b). In contrast, atrial myocytes correlated well with pacemaking myocytes and even more with non-pacemaking myocytes.

In conclusion, we successfully characterised the human primary pacemaker at single cell resolution by employing a novel A.I. deconvolution method. Our findings shed light on the biology of the human SN and its unique and compartmentalised cellular composition.

## Discussion

Bulk RNA-Seq has traditionally been used to study transcription patterns within tissues in a given biological state. Bulk transcriptomes, however, only provide an overall gene expression measurement from a mixture of cells and fail to identify the heterogeneous expression patterns associated with specific cell types living within the tissue. Using Bulk2Space as a state-of-the-art deep learning deconvolution method we have demonstrated how bulk transcriptomes can be resolved into biologically feasible single cells profiles. By lifting the costs associated with the experimental work necessary for scRNA-Seq, this method makes the study of single cell biology more easily accessible to biologists. Importantly, our results show that this method can be used to reveal the human cellular composition using data from mammalian non-human models such as mice, further expanding the use case of Bulk2space.

As a proof of concept, we chose to deconvolute an anatomical structure whose cellular landscape is well known. Thus, the human right atrium bulk transcriptome was deconvoluted using scRNA-Seq data from mice. We successfully recapitulated the cellular diversity of the human right atrium into eight cell types, with cardiac myocytes being the most abundant. As expected, we found a high degree of correlation in terms of gene expression between human and mouse cells. Further analysis of the eight atrial cell types revealed 18 cell subpopulations, highlighting the cellular heterogeneity of the atrium. The marker genes associated with each cluster and thus, cell type, were in line with other studies on cardiac cell populations^6,26–28^. This suggests that our single cell characterization of the human right atrium is biologically accurate and achievable using Bulk2Space as a novel deep learning-based deconvolution method. It should be noted that the choice of the reference dataset is pivotal when using this method. The biology of the bulk transcriptome must match as closely as possible that of the reference dataset on which the model is trained. Finding a biologically comparable reference dataset can be a challenging task due to the limited number of studies available on the tissue of interest.

Based on the results from the human right atrium, we employed this method to decipher the cellular landscape of the human SN. Despite being a small anatomical tissue, the human SN is characterised by a remarkable cellular complexity. Histologically, the SN contains small myocytes encapsulated in abundant ECM^4,9,29^. In line with such micro-anatomy, we showed that SN myocytes are the most abundant population of cells, followed by fibroblasts and adipocytes as the main cell populations of the ECM. We found that SN myocytes can be divided into pacemaking and non-pacemaking cells. While both expressed myocyte gene markers such as *MYH6, CTNNA3* and *TBX5*, pacemaking myocytes selectively expressed key ion channel genes such as *HCN1, HCN4, CACNA1D,* as well as transcription factors *SHOX2* and *TBX3*. Experimental characterization of pacemaking myocytes was performed in our previous work^4,29^. Importantly, the marker genes of pacemaking myocytes and fibroblasts were in line with those reported in another independent study of the human cardiac niches^9^, validating our results. Fibroblasts and adipocytes were the most abundant non-myocyte populations residing in the local connective tissue. Interestingly, fibroblasts of the human SN express a subset of collagen genes including *COL4A1, COL4A2, COL6A3* and *COL6A6*, which may provide unique mechanical properties to the ECM. The role of immune cells and resident macrophages is becoming increasingly more relevant in the electrophysiology of the SN^20,24^, suggesting that a diverse range of cell types play an important regulatory role in this tissue. In line with this, we have identified several subpopulations of lymphoid cells as well as macrophages within the human SN. Lastly, we showed that the human SN has a unique cellular composition. While the SN and the right atrium share several cell types, there is minimal correlation between the two. Conversely, we found a noticeable correlation between myocytes of the two tissues, with non-pacemaking myocytes being the most similar to atrial myocytes. This suggests that myocyte populations may be biologically stratified. Tissue-specific marker genes were experimentally validated by our group previously^20,23^. Based on this data, we speculate that the heartbeat originates in pacemaking myocytes, propagates to non-pacemaking myocytes within the SN, and finally reaches “peripheral” myocytes of the right atrium.

In conclusion, we successfully deciphered the cellular landscape of the human SN at single cell resolution, encompassing the heterogeneous biological complexity within the human primary pacemaker of the heart. Given the ever-growing amount of ‘omics data and the concurrent development of A.I. methods to support their analysis, it is inevitable that our understanding of biology will expand dramatically. This study provides the evidence of how A.I. can be a powerful tool to unravel the cellular architecture of the human SN in an unprecedented manner. Ultimately, this deconvolution method has the potential to be used in characterising the single cell composition of other components of the cardiac conduction system and other less defined human tissues.

## Methods

### Computing environment

Deconvolution and single cell analysis was performed on a machine with the following specifications: CPU (AMD Ryzen 5600X, 3.7 GHz × 6 cores), GPU (Nvidia RT 3070 Founders Edition, 8 GB VRAM), RAM (32 GB DDR4, 8 GB × 4, 3200 MHz), Storage (SSD NME M.2, 1 TB), Operating System (Windows 10 Pro, 22H2), Running environment (CUDA 11.8, Torch 1.12.1, Python 3.8.5, R 4.1.1, Visual Studio Code 1.82.2, RStudio 2023.06.1, Seurat 4.3.0., dplyr 1.1.2, deep-forest 0.1.5, easydict 1.9, numpy 1.19.2, pandas 1.1.3, scanpy 1.8.1, scikit-learn 1.0.1, scipy 1.5.2, tqdm 4.50.2, Unidecode 1.3.0).

### Data pre-processing

Prior deconvolution, bulk RNA-Seq data and all reference datasets were pre-processed as specified by the authors of Bulk2Space. Briefly, bulk RNA-Seq data was supplied to Bulk2Space as normalised read counts with at least 1 read, moreover duplicated and ambiguous genes were excluded; single cell reference data was supplied as two input files: a normalised count matrix with genes as rows and cells as columns, and an annotation file with “cell ID” and “celltype” columns. For the right atrium reference dataset, cells from the subtissue “RA” were selected in the normalised count matrix from the mouse heart of Tabula Muris. Similarly, cells belonging to the atria were selected from the normalised count matrix of Tabula Sapiens. Cells annotated as ventricular cardiomyocytes or hepatocytes were excluded as they do not belong in the atrium.

The optional spatial transcriptomics input files were not used as we focused on single cell analysis only. Thus, we supplied as empty count matrix and empty spatial coordinates. Detailed input data format can be found on the Github page of the authors of Bulk2Space (https://github.com/ZJUFanLab/bulk2space).

### Conversion of mouse genes into human genes

A collection of mouse-to-human orthologs was retrieved from Ensemble’s BioMart and used as a reference to convert mouse genes into their human counterpart. For simplicity, we converted genes that had a direct ortholog. Finally, we cleaned the converted count matrix by removing NaN and duplicated entries.

### Single cell deconvolution

Deconvolution was performed with the train_vae_and_generate method of Bulk2Space. Epoch_num was set to 3500, while other parameters were used as default. The top 200 marker genes of the newly generated human single cell data and mouse reference data were calculated by the ‘rank_genes_groups’ function in Scanpy. P value was calculated with the Wilcoxon rank-sum test. Pairwise correlation was calculated with using Pearson correlation coefficient.

### Single cell analysis

Deconvoluted scRNA-Seq data was pre-processed analysed using the Seurat 4.3.0^30^. For quality control, cells with more than 200 genes and genes detected in at least 3 cells were included in downstream analysis. Cells with a mitochondrial content over 25% were excluded. Highly variables genes were identified with FindVariableFeatures using the “vst” method on 2000 features. Data was scaled and linear dimension reduction was performed with RunPCA. The dimensionality of the dataset was calculated either with JackStraw or ElbowPlot. K-nearest neighbour was computed with FindNeighbors on 12 dimensions for both right atrium and SN datasets, followed by Louvian clustering with FindCluster. Resolution was set to 0.1 and 0.2 for the right atrium and SN datasets, respectively. Uniform Manifold Approximation and Projection (UMAP) and t-stochastic neighbour embedding (tSNE) were used to project the cells into a low dimensional space, with tSNE being chosen for better clustering results. Differentially expressed genes were calculated with FindAllMarkers, setting “min.pct” and “logfc.threshhold” to 0.25. Gene ontology enrichment analysis was performed with ShinyGO 0.77^31^.

### Single cell integration and comparative analysis

Integration of scRNA-Seq data was performed using Seurat 4.3.0^30^. Due to the presence of similar cell types across RA and SN, we chose a Canonical Correlation Analysis based integration approach. Batch effect artifacts were corrected through identification of common anchors across datasets^19,32^. Highly variable genes, scaling, dimensionality reduction, K-nearest neighbour and Louvian clustering were performed as described in the single cells analysis. Resolution was set to 0.2. The top 200 marker genes of right atrium and SN cells were calculated by the ‘rank_genes_groups’ function in Scanpy. P value was calculated with the Wilcoxon rank-sum test. Pairwise correlation was calculated with using Pearson correlation coefficient.

### Statistical analysis

All analyses were performed using R 4.1.1 and Python 3.8.5. Wilcoxon rank-sum test and Pearson correlation coefficient were used to determine differential gene expression and genetic similarity. *P* < 0.05 was considered statistically significant. Bonferroni correction was used for multiple testing.

### Supplementary Information

Supplementary Information.

## Data Availability

The human bulk RNA-Seq data of the right atrium and SN has been previously described^20^ and is available upon request. Deconvoluted count matrices of the human SN and RA can be accessed at: https://figshare.com/articles/dataset/Deconvoluted_scRNA-seq_datasets/25605297. The mouse scRNA-Seq data used as reference for the right atrium can be accessed at: https://cellxgene.cziscience.com/collections/0b9d8a04-bb9d-44da-aa27-705bb65b54eb. The human scRNA-Seq data used as reference for the right atrium can be accessed at: https://figshare.com/articles/dataset/Tabula_Sapiens_release_1_0/14267219?file=34701976. The mouse snRNA-Seq data used as reference for the SN can be accessed from the Gene Expression Omnibus under the “GSE130710” accession code.

## Code availability

All code used for this study can be found in the author’s Github page: https://github.com/AlexUOM/Sinus-node-deconvolution.
