# Supplementary material for: Empowering artificial intelligence in characterizing the human primary pacemaker of the heart at single cell resolution
Source: Sci Rep. 2024 Jun 18;14:14041. doi: 10.1038/s41598-024-63542-6 (PMC11189420; doi:10.1038/s41598-024-63542-6)
Supplement: Supplementary file 1 — Supplementary Information. [file 41598_2024_63542_MOESM1_ESM.docx]

**Supplementary Information**

***Empowering artificial intelligence in characterizing the human primary pacemaker of the heart at single cell resolution***

Alexandru Chelu^1^*, Elizabeth J Cartwright^1^, Halina Dobrzynski^1-2^

^1^Division of Cardiovascular Sciences, Faculty of Biology, Medicine and Health, The University of Manchester, M13 9PL Manchester, United Kingdom.

^2^Department of Anatomy, Jagiellonian University Medical College, 31-008 Krakow, Poland.

**Contents**

Supplementary Data 1

**
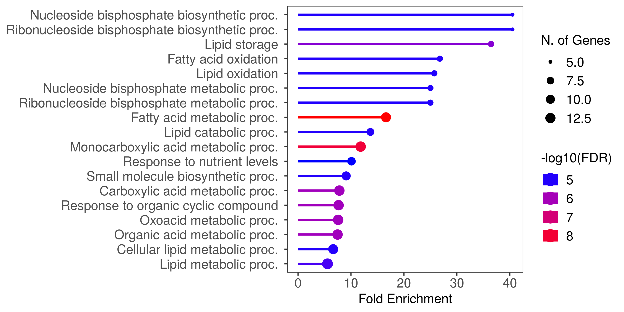
**
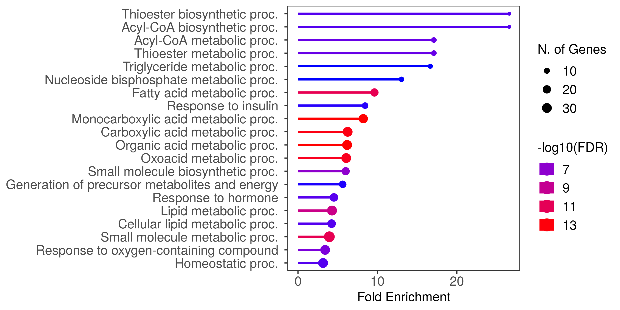


**a**

**b**

**Adipocytes 2**

**Adipocytes 1**

**Macrophages**

**c**

**d**

**Fibroblasts**

**
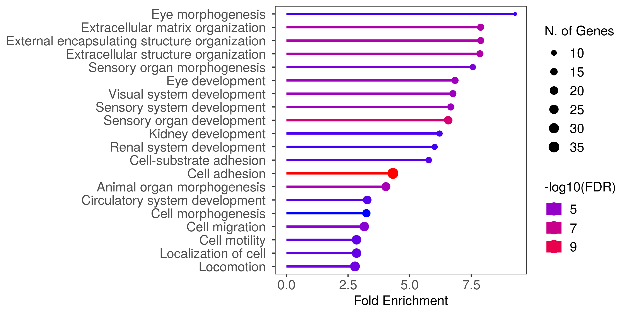

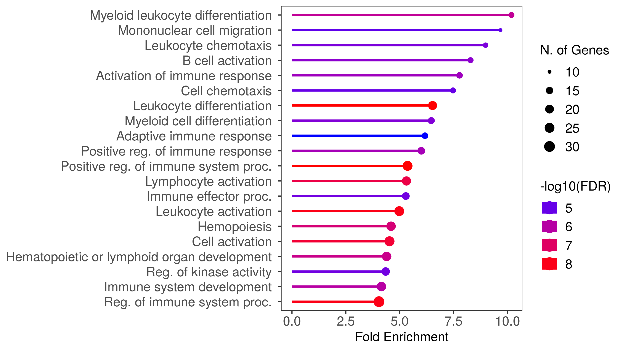
**

**e**

**Lymphoid cells 2**

**Lymphoid cells 1**

**Lymphoid cells 1**

**f**

**
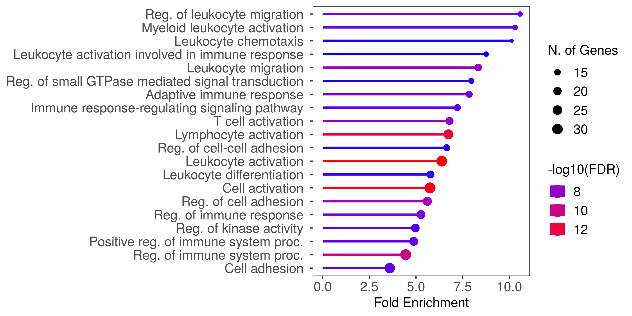

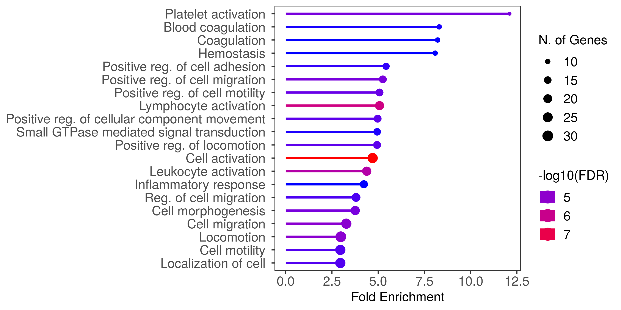
**

**Lymphoid cells 3**

**g**

**
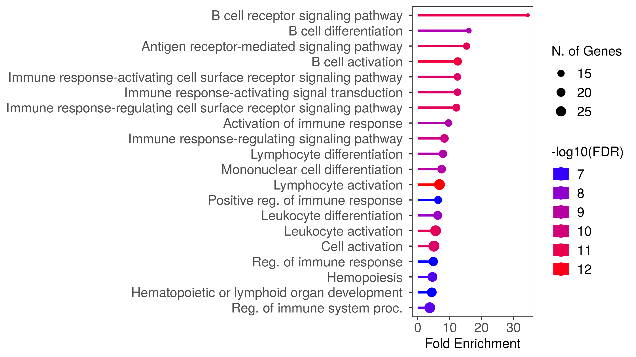
**

**Supplementary Data 1. Enrichment analysis of non-myocytes.** **a-g** Lollipop plots showing the significantly enriched biological processes in non-myocyte cells populations according to Gene Ontology.
